# Prevalence and Risk Factors of Home Accidents Among Children Under Five Years of Age in Al-Baha, Saudi Arabia

**DOI:** 10.7759/cureus.46846

**Published:** 2023-10-11

**Authors:** Fahad Alamr, Hadeel Mohammed A Alzahrani, Ahmed Mohammed A Alghamdi, Abdulrhman Saleh A Alzhrani, Feras Atiyah A Alzahrani, Lama Mohammad A Alkhediwi, Mohammed Ahmed A Alghamdi, Meelaf Ali M Alhomrani, Omer M Aburaida

**Affiliations:** 1 College of Medicine, Al-Baha University, Al-Baha, SAU; 2 Paediatrics and Child Health, College of Medicine, Al-Baha University, Al-Baha, SAU

**Keywords:** trauma pediatric, risk factors, prevalence, children, home accidents

## Abstract

Introduction: Internationally, home accidents are the main cause of preventable debilities and death among children and young persons. Many times, children survive accidents with physical or mental damage that curtails their activities in the long term. The most commonly reported accidental injuries include head injuries, open wounds, and poisoning. This study aims to assess the prevalence and factors associated with home accidents among children under five years old in the Al-Baha region, Saudi Arabia.

Methods: A descriptive cross-sectional study was conducted among the community population in the Al-Baha region, Saudi Arabia, targeting all accessible parents who have children under five years old. A convenience sampling technique was used for sample collection during the period of three months (May 2023 to July 2023), where all accessible parents who fulfilled the inclusion criteria and agreed to participate were invited to fill out the received online study questionnaire. Section 1 covered the participants' demographic data. The second section covered the children’s data and the third section included questions about home accident types, frequency, severity, and causes.

Results: The findings showed that 205 (58.2%) study parents reported a history of home accidents among their children. As for accident data, about 122 (59.5%) of the injured children were males. The most reported home accidents among children were fall/impact with hard objects (58.2%), burn (30.7%), asphyxia (27.6%), and poisoning (24.4%). Families with more than seven members and those with four or more siblings significantly experienced higher home accidents than others (p<0.001).

Conclusion: In conclusion, the current study showed that home accidents among children under five years of age were mainly falls and burns; they were mainly found among male children and children in families with highly educated mothers and many kids. A majority of the reported cases of home accidents were less severe and the hospitalization rates with complications were very few.

## Introduction

Home accidents (HAs) are becoming a significant contributor to childhood traumatic injuries and long-term disability, yet they are still underreported and underestimated [1]. As a matter of public health, child injuries sustained in the home are a concern. Globally, an annual estimated one million child fatalities and 10 million child injuries are attributed to HA [2]. In the realm of household accidents, where newborn fatalities occur, there are abundant cases of nonfatal injuries, each presenting a diverse range of morbidity levels. Most statistics relate the home environment to child accidents, and research shows that the HA index is consistently greater than 50% [3]. There is a lack of breakdown data on HAs in Saudi Arabia by province [4]. According to a review of the epidemiology of accidental child injuries undertaken in five countries (Bangladesh, Colombia, Egypt, Malaysia, and Pakistan), 56.8% of child injuries were from HAs [5]. Research undertaken in 16 European nations discovered an association between the home environment and the probability of accidents involving children less than five years old [6].

HAs related to foreign bodies in orifice/ingestion were highest among toddlers and preschoolers, where a strong association between decreased age and incidence of HA was observed, which might be due to the development of children’s cognitive abilities with age [7]. Evidence indicates that male children experienced significantly higher rates of HAs than females [8].

Socioeconomic and environmental factors influence the incidence of HA. It is possible that the child, his/her material surroundings, or the items involved in the accidents, all played a role in bringing about the accident [9]. Surviving an accident is no guarantee that a child will not have permanent mental or physical scars that may limit the child's future opportunities. Poisoning, head injuries, and open wounds are the most often reported HAs. Accident injuries are costly to treat, both for healthcare providers and the victims' families, even when they don't persist for a long time [10]. Since there is a lack of information on HA in Saudi children, this study was designed to uncover the prevalence of HA and the associated risk factors among children less than five years old in the Al-Baha region of Saudi Arabia.

## Materials and methods

A descriptive cross-sectional study was conducted among the general population in the Al-Baha region, Saudi Arabia, and targeted parents who have children under five years old. Parents outside the Al-Baha region, parents who don’t have children aged <5 years, and parents who refused to participate in the study were excluded. Participants were recruited from hospitals and primary health centers where a mixture of snowball and convenience sampling was employed. We recruited the participants till we achieved the minimum sample size with an additional 15% beyond the minimum sample size as a precautionary measure to account for unexpected issues that may reduce the effective sample size, such as dropouts or incomplete data.

The minimum sample size was calculated based on the values obtained from a pilot study on 20 parents and was found to be 329.

n=Z^2^p(1 - p)/ME^2^,

where n is the required sample size, Z corresponds to the chosen confidence level of 95% (Z ≈ 1.96), p is the expected prevalence rate (p = 0.31), and ME is the margin of error (5%).

Data were collected through an online structured questionnaire. The questionnaire was pretested on 20 participants and it showed acceptable reliability (Cronbach's alpha = 0.706). Face validity was assessed by a focused group discussion conducted by three experts (a consultant in community medicine, a consultant in pediatrics, and a biostatistician). Content and criterion-related validity were not done due to paucity of time. Modifications were done as per the suggestion of each expert, and the final questionnaire was drafted. The original version of the questionnaire was developed in English language and then translated into an Arabic version by an expert who is proficient in both languages. The Arabic version was then back-translated to English by another expert who was blinded to the original English version.

The data were collected using an online Arabic version (Google Forms) and were distributed via multiple social media applications (mainly through WhatsApp, Snapchat, and Telegram). The assurance of data confidentiality was meticulously upheld throughout the study. All personal and sensitive information provided by the respondents, including demographic details and questionnaire responses, were treated with utmost confidentiality. Participant anonymity was maintained by assigning unique identification codes dissociated from personal identifiers. Ethical research practices were observed by all personnel, including five data collectors and one analyst, who underwent training in confidentiality protocols. These measures collectively ensured the protection of participant information throughout the research process.

The final version of the questionnaire included 25 items divided into three main sections. Section 1 covered participants' demographic data. The second section covered the children's data, and the third section included questions about HA types, frequency, perceived severity of injury (5-point Likert scale), and causes. The prevalence of HAs was calculated as the proportion of children who had experienced at least one HA within the past year, divided by the total number of participants. Ethical approval was obtained from the Institutional Review Board of Al-Baha University.

Data analysis

After data were extracted, they were revised, coded, and fed to statistical software IBM SPSS version 22 (IBM Corp, Armonk, NY). Descriptive analysis based on frequency and percent distribution was done for all variables: parents' demographic data, education, monthly income, family size, and sibling's number. Also, types, frequency, severity, causes, and children-related data for HAs were tabulated and graphed. Cross-tabulation was used to assess factors associated with HAs among children less than five years old and was tested using Pearson's chi-square test and exact probability test for small frequency distributions. All statistical analyses were done using two-tailed tests, and a p-value less than 0.05 was considered statistically significant.

## Results

A total of 352 eligible participants completed the study questionnaire. The sociodemographic analysis showed that about 173 children (49.1%) had a family size of 5-7, 203 (57.7%) had more than four siblings, and 328 (93.2%) had both parents alive. Other characteristics are given in Table 1.

**Table 1:** Family characteristics of study respondents, Al-Baha region, Saudi Arabia

Family characteristics	No.	%
Family size		
3-4	130	36.9
5-7	173	49.1
>7	49	13.9
Siblings		
1	33	9.4
2	92	26.1
3	24	6.8
4+	203	57.7
Parents are alive		
Yes, both	328	93.2
Only mother	19	5.4
Only father	5	1.4
Parents divorced		
Yes	26	7.4
No	326	92.6
Mother work		
Housewife	196	55.7
Working	156	44.3
Mother education		
Below secondary	43	12.2
Secondary	77	21.9
University/postgraduate	232	65.9
Father education		
Below secondary	22	6.3
Secondary	82	23.3
University/postgraduate	248	70.5
Monthly income (in Saudi Riyals)		
<5000	32	9.1
5000-10,000	114	32.4
>10,000	206	58.5
There is another caregiver for a child other than the parents, a maid or servant		
Yes	103	29.3
No	249	70.7
Home		
Owned	267	75.9
Rented	85	24.1
Age of the parent participated		
≤25 years	142	40.3
26-35 years	127	36.1
36-45 years	54	15.3
46-55 years	23	6.5
≥56 years	6	1.7

About 205 (58.2%) participants reported the prevalence of HA in their children as for accident data, and exactly 122 (59.5%) of the injured children were males. As for age, 21 (10.2%) were aged less than one year, 98 (47.8%) were aged 1-3 years, and 86 (34.1%) were aged 4-5 years. Exactly 50 (24.5%) experienced accident-related complications and 52 (25.5%) were hospitalized. A total of 51 (24.9%) were the first child (Table 2).

**Table 2:** Frequency of home accidents among children aged 1-5 years (n = 205)

Home accident data (n = 205)	No.	%
Gender of injured child		
Male	122	59.5
Female	83	40.5
Age of the injured child (n = 205)		
<1 year	21	10.2
1-3 years	98	47.8
4-6 years	86	42
Are there any complications due to the accident?		
Yes	50	24.5
No	154	75.5
Was hospitalized due to an accident		
Yes	52	25.5
No	152	74.5
Is (he/she) the first child in the family		
Yes	51	24.9
No	154	75.1

**Figure 1:**
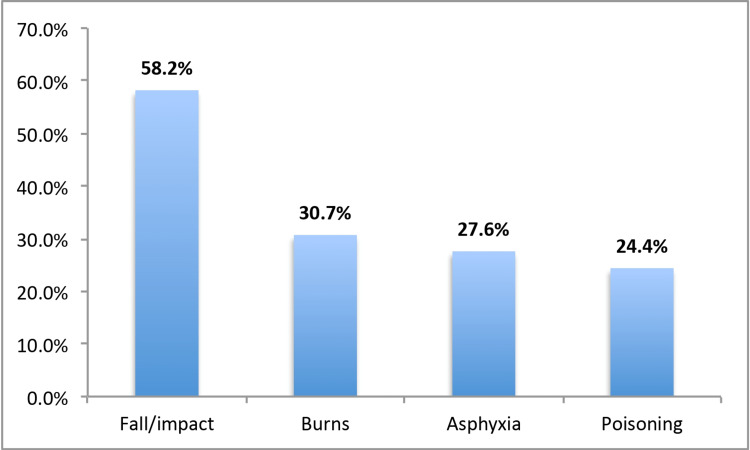
Types of home accidents among children under five years of age in the Al-Baha region

Figure 1 shows the types of HAs among children under five years of age in the Al-Baha region. The most reported HAs among children were fall/impact with hard objects (58.2%), burn (30.7%), asphyxia (27.6%), and poisoning (24.4%). 

Table 3 lists the types, frequency, severity, and causes of HAs in Al-Baha, Saudi Arabia. A total of 20.5% of children who had fallen had lost consciousness and 50% had fallen for one time. Injury severity was assessed using a 5-point Likert scale (1 - mild to 5 - very severe) and it was found that about 9.2% gave a score of 5 and 11.2% a score of 4. Asphyxia was due to airway obstruction among 55.7% of the cases and due to accidental strangulation by hand/rope while playing among 16.5%. The severity score was 4 to 5 among 10.4%. Burns were due to liquid substances in 41.7% of the children and due to fire in 42 (38.9%). Considering the burn site, it was at the upper extremities in 61.5% of the children, lower extremities in 22%, and in the abdomen in 9.9%. About 24.4% had reported poisoning and the most commonly reported was accidental bite by an insect/animal (22.1%), followed by ingestion of chemicals (16.3%) and drugs (9.3%), and the remaining were accidental poisoning by other variant materials. As for actions taken with poisoning, the most reported were going to the hospital (48.3%), calling an ambulance (15%), and applying first aid (3.3%), while 25% did nothing. 

**Table 3:** Types, frequency, severity, and causes of home accidents in Al-Baha, Saudi Arabia ^†^Cigarette lighter, matches, and candles; ^‡^household products, household plants, batteries.

Home accident types/frequency		No.	%
Fall/impact	Yes	205	58.2
	No	147	41.8
Did the child lose consciousness after the fall or impact?	Yes	42	20.5
	No	163	79.5
Number of accidents	1	76	50.0
	2	40	26.3
	3	36	23.7
Injury severity, where 5 is the highest and 1 is the lowest	1	38	25.0
	2	42	27.6
	3	41	27.0
	4	17	11.2
	5	14	9.2
Asphyxia	Yes	97	27.6
	No	255	72.4
Causes of asphyxia	Airway obstruction	54	55.7
	Have chemical material	13	13.4
	Drowning	7	7.2
	Accidental strangulation by hand or rope (while playing)	6	6.2
	A fire	16	16.5
	Toxic gas	1	1.0
Number of asphyxias	1	79	81.4
	2	13	13.4
	3	5	5.2
Injury severity, where 5 is the highest and 1 is the lowest	1	43	44.3
	2	20	20.6
	3	21	21.6
	4	8	8.2
	5	5	5.2
Burn	Yes	108	30.7
	No	244	69.3
Causes of burn	Incendiary substance^†^	21	19.4
	Liquid substance	45	41.7
	Fire	42	38.9
Number of burns	1	74	73.3
	2	18	17.8
	3	9	8.9
Site of burn	Upper extremities	56	61.5
	Lower extremities	20	22.0
	Abdomen	9	9.9
	Chest	3	3.3
	Face	3	3.3
Poisoning	Yes	86	24.4
	No	266	75.6
Poison (n = 86)	Chemicals (bleach, drain cleaner, or detergents)	14	16.3
	Accidental insect or animal bite	19	22.1
	Drugs	8	9.3
	Others^‡^	45	52.3
Number of poisonings	1	40	81.6
	2	7	14.3
	3	2	4.1
How to deal with an injury when it occurs	Nothing	15	25.0
	Call ambulance	9	15.0
	First aids	2	3.3
	Go to hospital	29	48.3
	Others	5	8.3

Factors associated with HAs among children are given in Table 4. A total of 81.6% of children with large family sizes (>7) experienced HAs compared to 48.5% of children with family sizes 3-4 persons with recorded statistical significance (p = 0.001). Also, 64.5% of children with four or more siblings had HAs versus 39.4% of children with no siblings (p = 0.021). HAs were reported among 64.6% of children with secondary educated fathers compared to 31.8% of others with less educated fathers (p = 0.021). Additionally, 70.9% of children with another caregiver experienced HAs compared to 53% of those who were cared for by their parents (p = 0.002). 

**Table 4:** Factors associated with home accidents among children under five years of age in the Al-Baha region *A p-value <0.05 is considered statistically significant.

Factors	Has the child been exposed to accidents at home?				p-Value
	Yes		No		
	No.	%	No.	%	
Family size					0.001*
3-4	63	48.5%	67	51.5%	
5-7	102	59.0%	71	41.0%	
>7	40	81.6%	9	18.4%	
Siblings					0.021*
1	13	39.4%	20	60.6%	
2	49	53.3%	43	46.7%	
3	12	50.0%	12	50.0%	
4+	131	64.5%	72	35.5%	
Parents are alive					0.385
Yes, both	192	58.5%	136	41.5%	
Only mother	9	47.4%	10	52.6%	
Only father	4	80.0%	1	20.0%	
Parents divorced					0.723
Yes	16	61.5%	10	38.5%	
No	189	58.0%	137	42.0%	
Mother work					0.181
Housewife	108	55.1%	88	44.9%	
Working	97	62.2%	59	37.8%	
Mother education					0.184
Below secondary	29	67.4%	14	32.6%	
Secondary	39	50.6%	38	49.4%	
University/postgraduate	137	59.1%	95	40.9%	
Father education					0.021*
Below secondary	7	31.8%	15	68.2%	
Secondary	53	64.6%	29	35.4%	
University/postgraduate	145	58.5%	103	41.5%	
Monthly income (in Saudi Riyals)					0.858
<5000	19	59.4%	13	40.6%	
5000-10,000	64	56.1%	50	43.9%	
>10,000	122	59.2%	84	40.8%	
There is another caregiver for a child other than the parents, a maid or servant					0.002*
Yes	73	70.9%	30	29.1%	
No	132	53.0%	117	47.0%	

## Discussion

HAs among children are a significant concern as they can result in injuries or even fatalities. Fire, burns, suffocation, drowning, choking, falls, poisoning, and gun accidents are common HAs [11]. Kitchens, bathrooms, swimming pools, hot tubs, barbecue grills, and other heat sources are common places for these accidents [12-14]. The current findings showed that the prevalence of HAs among children was high. Reports from the USA showed that domestic violence claims the lives of 2,800 American children annually, with an additional 13 million requiring at least one outpatient treatment and 74,000 requiring hospitalization [15]. A retrospective study in the Sultanate of Oman reported a prevalence of 7.7% of HAs among children <18 years of age who were presented to the emergency department of a university hospital [16]. The prevalence reported in our study is a little higher compared to another study done in the Qassim region, which reported a prevalence of 46.3% among 250 participants [17]. Falling, burns, cuts, choking, and poisoning were common causes of HAs among children. This is much lower than that reported in the current study. Also, a lower incidence was reported in previous studies and the reports of childhood injury surveillance in Egypt and São Paulo in Brazil [18,19]. A bit higher incidence was reported among Swedish teenagers, where HAs contributed to 26% of all accidents in the age group 0-19 years and about 10% of all healthcare consumption, where 10% were hospitalized [20]. 

Tenure of the household was the only factor that showed a significant relation to HAs. Murdock et al. documented that 74·5% of accidents among children were to children under five years of age [21]. Boys were more prone to accidents than girls, and in preschool children, the highest incidence of accidents was among the two- to three-year-olds of both sexes. Locally, Ghailan K et al. in Jazan found that the incidence rate of child home incidents was 7.4 per 100 children in 2018 [22]. Falling, burning, swallowing foreign bodies, and domestic violence were the most frequent types of injuries reported. Home injuries in one year included 36.8% bone fractures, 31.6% body distortions, 9.2% distortion fractures, and 5.3% child impairment. The most commonly reported type of HA in our study was fall, and this is similar to the findings of Albedewi H et al., which found a prevalence of 31.9% HA due to fall and 25.1% due to motor vehicle collision in the Jazan region, Saudi Arabia [23]. The same study also reported a weighted mortality rate of 5.2% for overall injuries, 8.3% for fractures of the skull and spine, and 17.4% for burns. Moreover, other studies in the Middle Eastern countries have reported similar types of HA-related injuries but with a different trend in terms of prevalence [4,18,24]. Preventing small children from falling from heights at home requires a combination of safety measures and an understanding of the science behind child development and injury prevention. Safety gates are effective tools for preventing young children from accessing staircases or areas with height differences [25]. Securing heavy furniture to the wall with safety straps or anchors prevents tip-over accidents, addresses the biomechanics of child movement, and prevents the exertion of force that could topple furniture [26]. Young children have limited impulse control and may not fully grasp the consequences of their actions [27]. Constant supervision allows caregivers to intervene immediately if a child attempts to climb, lean over railings, or engage in risky behavior near heights [28].

As for risk factors analysis, the current study showed that a high accident rate among children was associated with having four or more children, a large family size, high mothers' education (mostly working), and having another caregiver for a child other than the parents, a maid or servant. Other studies showed that risk factors for HAs among children include lack of parental education and awareness, inadequate supervision, unhealthy home conditions such as poor ventilation, and the presence of hazardous objects or substances at home [3,29]. Large families with four or more children may have a higher risk of HAs because there are more children present, increasing the likelihood of accidents [30]. Large families may also have more items and activities at home, potentially leading to more opportunities for accidents [31]. While larger families may inherently have a higher accident rate due to the number of children, it is important to delve deeper into specific contributing factors within these families, such as supervision levels and safety measures. Mothers with higher education levels who are mostly working may have busy schedules and less time for direct child supervision [32]. This can lead to an increased risk of accidents, especially if children are left unsupervised for extended periods. The presence of alternative caregivers, such as maids or servants, can have both positive and negative effects on child safety. While they can provide additional supervision, the quality of care and safety practices of these caregivers may vary, potentially impacting child safety [33]. Proper training and clear safety guidelines can mitigate risks associated with alternative caregivers. At the same time, it is important to differentiate between correlation and causation. While these factors are associated with higher accident rates, other variables such as the home environment, child behavior, and parental knowledge also play critical roles. To address these findings effectively, further research is needed to explore the nuanced relationships among these factors. It is essential to consider the quality of supervision, the implementation of safety measures within the home, and the role of education and awareness in mitigating accident risks. Tailored interventions, such as safety education for parents and alternative caregivers, childproofing measures, and policies that support working parents, may help reduce HAs among children in these circumstances [34,35].

Another significant finding of our study is that about 28.4% of the parents reported that their child had repeated poisoning two or more times. The repeated nature of poisoning incidents indicates that there may be a lack of awareness or education among parents about poison prevention [36]. Launching comprehensive public awareness campaigns targeting parents, caregivers, and the general population could help to mitigate this lack of awareness. Additionally, integrating poison prevention education into the school curriculum at various levels and offering parenting classes and workshops that cover topics related to child safety, including poison prevention, could be beneficial [37]. It is also important to assess the level of parental knowledge regarding household poisons, safe storage, and the importance of keeping toxic substances out of the reach of children. Furthermore, the findings showed that one-fourth of the participants did nothing in case of injury. One possible justification is that some participants may lack the necessary knowledge and skills to respond effectively to child injuries [38]. Also, it could be due to poor awareness regarding the appropriate first-aid measures or due to misconceptions about what to do in such situations [39]. In the event of a child's injury, especially if it is a severe or unexpected accident, panic can overwhelm caregivers, and this may impair judgment and decision-making, leading to inaction. Some individuals may worry that their actions could exacerbate the child's injury, leading them to hesitate or refrain from taking immediate action [40].

Several limitations were acknowledged in the course of this cross-sectional study. First, the sample size in our study is small and may not accurately reflect the prevalence of HAs in the entire population, leading to potential generalizability issues. Secondly, the study's cross-sectional design restricted the establishment of causal relationships between risk factors and HAs, as it captured data at a single point in time. This design also made it susceptible to recall bias, as participants were required to recollect past incidents. Additionally, the study's reliance on self-reported data from parents or guardians might have introduced reporting bias or subjective interpretations. The use of an online questionnaire, although convenient for data collection, could have excluded certain segments of the population without internet access or digital literacy, potentially leading to sample bias. Furthermore, the generalizability of the findings beyond the studied region might be limited due to the specific sociodemographic characteristics of Al-Baha.

## Conclusions

In conclusion, the research highlights that HAs among children under five years of age, particularly falls and burns, are relatively frequent occurrences. The study shows a notable association with male children, highly educated mothers, and families with multiple siblings. Despite the frequency of these accidents, the severity rate remains relatively low, and instances of hospitalization due to injuries are uncommon, with fewer complications observed. The findings could guide healthcare professionals, parents, and caregivers in implementing targeted preventive measures to reduce the occurrence of falls and burns, especially in households with educated mothers and larger families. By identifying the relatively low severity and hospitalization rates, healthcare resources can be directed toward more critical cases, while non-severe accidents can potentially be managed at home with appropriate guidance. Overall, this research underscores the importance of continued vigilance and education about child safety in the home environment. It is important for parents and caregivers to be aware of the potential risks and take necessary precautions to create a safe home environment for children.
